# Direct Chromatin PCR (DC-PCR): Hypotonic Conditions Allow Differentiation of Chromatin States during Thermal Cycling

**DOI:** 10.1371/journal.pone.0044690

**Published:** 2012-09-12

**Authors:** Sergei Vatolin, Shahper N. Khan, Frederic J. Reu

**Affiliations:** 1 Department of Translational Hematology and Oncology Research, Taussig Cancer Institute, The Cleveland Clinic Foundation, Cleveland, Ohio, United States of America; 2 Department of Hematologic Oncology and Blood Disorders, Taussig Cancer Institute, The Cleveland Clinic Foundation, Cleveland, Ohio, United States of America; University of North Carolina at Chapel Hill, United States of America

## Abstract

Current methods to study chromatin configuration are not well suited for high throughput drug screening since they require large cell numbers and multiple experimental steps that include centrifugation for isolation of nuclei or DNA. Here we show that site specific chromatin analysis can be achieved in one step by simply performing direct chromatin PCR (DC-PCR) on cells. The basic underlying observation was that standard hypotonic PCR buffers prevent global cellular chromatin solubilization during thermal cycling while more loosely organized chromatin can be amplified. Despite repeated heating to >90°C, 41 of 61 tested 5′ sequences of silenced genes (CDKN2A, PU.1, IRF4, FOSB, CD34) were not amplifiable while 47 could be amplified from expressing cells. Two gene regions (IRF4, FOSB) even required pre-heating of cells in isotonic media to allow this differentiation; otherwise none of 19 assayed sequences yielded PCR products. Cells with baseline expression or epigenetic reactivation gave similar DC-PCR results. Silencing during differentiation of CD34 positive cord blood cells closed respective chromatin while treatment of myeloma cells with an IRF4 transcriptional inhibitor opened a site to DC-PCR that was occupied by RNA polymerase II and NFκB as determined by ChIP. Translation into real-time PCR can not be achieved with commercial real-time PCR buffers which potently open chromatin, but even with simple ethidium bromide addition to standard PCR mastermix we were able to identify hits in small molecules screens that suppressed IRF4 expression or reactivated CDKN2A in myeloma cells using densitometry or visual inspection of PCR plates under UV light. While need in drug development inspired this work, application to genome-wide analysis appears feasible using phi29 for selective amplification of open cellular chromatin followed by library construction from supernatants since such supernatants yielded similar results as gene specific DC-PCR.

## Introduction

The epigenetic activity of genes is determined by the net balance of activating and repressing histone modifications, methylation of regulatory CpG islands and binding of regulatory proteins and RNAs [1]. Mutations and environmental factors can affect epigenetic regulation via aberrant silencing or de-repression of genes contribute to disease, especially cancer but likely many other diseases including auto-immune disorders, cardiovascular disease, and diseases affecting the central nervous system [2], [3], [4]. In addition, certain cancers can become addicted to genes that are activated during maturation of their cells of origin and if expression is limited to few cell types such genes also may become attractive targets for chromatin or transcription modification. The transcription factor IRF4, highly and constantly expressed in antibody producing plasma cells and their malignant counterpart multiple myeloma but otherwise limited to lymphoid and myeloid cells where it is mostly expressed in response to stimuli, is such an example [5].

Our laboratory aims at identifying compounds that are able to activate or repress pathogenetically relevant chromatin sites through novel pathways. Reported methods for direct analysis of chromatin states include salt fractionation of micrococcal nuclease digested nuclei which yields active chromatin in the low salt (80–150 mM NaCl) and the salt insoluble fraction, whereas repressed chromatin can be found in the higher salt concentration fraction (600 mM NaCl). Salt insoluble areas of the genome were found to be in areas were RNA polymerase II and high-molecular-weight proteins interacted with DNA, apparently rendering it insoluble [6]. Although it offers the advantage over ChIP that it does not require assessment of activating and repressing modifications to assess net chromatin activity, it, like ChIP, is done on nuclear extracts posing a major challenge to high throughput drug screening applications. Nucleosome-free regions are enriched in active chromatin and susceptible to enzymatic cleavage but protocols also require nuclear extraction [7], [8]. Another way to obtain nucleosome-free DNA is by crosslinking histone-bound DNA followed by mechanical fragmentation through sonication [9] but here separation of DNA fragments makes centrifugation necessary and application in drug screens cumbersome. Therefore, we explored ways to investigate chromatin solubility without the need for centrifugation and found that the simplest method was to submit whole cells to direct chromatin-PCR (DC-PCR). Key to the success of this procedure is the observation that standard hypotonic PCR buffers prevent universal heat solubilization of cellular chromatin during PCR while areas with weaker DNA-protein bonds can be amplified.

## Materials and Methods

### Cell Culture

The multiple myeloma cell line RPMI 8226, acute myeloid leukemia (AML) cell lines MOLM-13, KG-1, and the erythroleukemia subtype of AML K562 were obtained from ATCC. Normal human adult dermal fibroblasts were purchased from Lonza. Multiple myeloma cell lines KMS-12-PE and KMS-12-BM and the secondary AML cell line SKM-1 were purchased from the JCRB. KMS-12-PE-luc was generated in our laboratory by transduction of KMS-12-PE with a Cignal ™ firefly luciferase control lentivirus obtained from Qiagen ™. Cord blood was obtained from Cleveland Cord Blood Center. Cells obtained from cord blood were kept in IMDM supplemented with 10% fetal bovine serum, penicillin G (50 units/ml), streptomycin (50 units/ml), and 10 ng/ml of the following cytokines, obtained from PeproTech: Interleukin-3, interleukin-6, stem cell factor, and FLT3 ligand. All other cells were kept in RPMI 1640 (NaCl 103.45 mM, NaCO_3_ 23.81 mM, Na_2_HPO_4_ 5.63 mM, KCl 5.33 mM, Ca(NO_3_)_2_ 4H_2_O 0.424 mM, MgSO_4_ 0.407 mM, pH around 7.2), supplemented with 10% fetal bovine serum, penicillin G (50 units/ml), and streptomycin (50 units/ml). All cells were cultured at 37°C, 5% CO_2_, and humidified air. Cells were counted and viability assessed using the Vi-CELL counter from Beckman Coulter, Inc. which uses trypan blue exclusion. All experiments were performed under conditions that yielded at least 85–95% viability in untreated cells. Decitabine (DAC) and Trichostatin A (TSA) were purchased from Sigma-Aldrich Co. LLC. Cells were incubated with these drugs at indicated concentrations and for indicated amounts of time.

### Direct Chromatin - PCR (DC-PCR)

1 µl of exponentially growing cells (from 1 to 1000 cells per reaction), de-attached from a plate by treatment with 0.25% trypsin –2.21 mM EDTA mix if adherently growing, were diluted directly into 25 µl of room temperature PCR mix (CDKN2A, PU.1, CD34, c-MYC) or after preheating in tissue culture medium to 80°C for 60 sec (IRF4, FOSB). PCR mix composition: 10 mM Tris-HCl, 50 mM KCl, 1.5 mM MgCl_2_, 200 µM dNTP, 1 U Taq-polymerase (New England Biolabs Inc.), 0.2 µM of corresponding forward and reverse primers, pH 8.3, supplemented with 1x Proteinase inhibitor cocktail, Set #5 (EMD Chemicals, Inc.) and 1 µM ethidium bromide (Sigma-Aldrich Co. LLC.). Amplification was started after incubation of cells in hypotonic PCR solution for 15–25 minutes at room temperature (21–23°C). PCR was run for 40 cycles in PCR cycler (Eppendorf, Mastercycler pro-S). To determine primer quality, genomic DNA was used. PCR conditions were: CDKN2A, (94°C, 30 sec; 64°C, 30 sec; 72°C, 30 sec)x40 → 72°C, 1 min. → 4°C, hold; CD34, (94°C, 60 sec; 60°C, 60 sec; 72°C, 60 sec)x40 → 72°C, 1 min. → 4°C, hold; FOSB, 94°C, 3 min. → (94°C, 60 sec; 64°C, 60 sec; 72°C, 60 sec)x40 → 72°C, 1 min. → 4°C, hold; IRF4 and PU.1, 94°C, 5 min. → (94°C, 30 sec; 58°C, 30 sec; 72°C, 30 sec)x40 → 72°C, 1 min. → 4°C, hold; cMYC: (94°C, 30 sec; 58°C, 30 sec; 72°C, 30 sec)x40–72°C, 60 sec –4°C, hold. Primer sequences, location and predicted sizes of PCR fragments are in Table S3. PCR products were analyzed by agarose gel electrophoresis and by direct UV transillumination in PCR multi-well plates. The relative DNA concentration after staining with ethidium bromide was read and quantified on Wallac 1420 Workstation plate reader (Tables S1 and S2). The identity of PCR fragments was verified by sequencing. The images of PCR plates were taken under a standard UV-light trans-illuminator. PCR products were resolved on 2% agarose gel prepared on 1x TBE buffer with 1 µM of ethidium bromide and also visualized under UV-light.

### Amplification of Open Chromatin using Phi29 Polymerase

For amplification of open chromatin using phi29 polymerase a REPLI-g kit (Qiagen) was used according to the manufacturer’s protocol with the following modification: to achieve selective amplification of open chromatin, the chemical denaturation step was skipped. 5 µL of cell suspension (1000 cells/µL) prepared as for gene-specific DC-PCR (with or without heating to 80°C for 60 sec for later assessment of FOSB and CDKN2A sequences, respectively) were placed into 45 µL of phi29 buffer (REPLI-g reaction buffer) supplemented with phi29 polymerase. The reaction was run for 4.5 hours at 30°C. After that, samples were centrifuged at 13000 rpm, for 10 min at 4°C on a tabletop Eppendorf centrifuge to pellet down cell carcasses. The upper 25 µL of the supernatant was placed in a clean tube and used to confirm preferential amplification of open chromatin sites in CDKN2A and FOSB genes. To do so, 1 µl of this upper fraction per primer pair was re-amplified by conventional PCR with Taq polymerase. PCR conditions are described in section for DC-PCR of Material and Methods. Primer sequences, location and predicted sizes of PCR fragments are in Table S3.

### Compound Library

The library of 5120 diverse chemical molecules was purchased from the Drug Development Center of the University of Cincinnati.

### DC-PCR based Small Molecule Screens

200 µl of cell suspension at 200,000 cells/ml in the 6 h incubation IRF4 screen and at 100,000 cells/ml in the 3 day incubation CDKN2A screen were treated with compound library in 96-well plates. Four wells were left completely untreated; four wells were treated with DMSO vehicle at final concentration of 0.2%. In the IRF4 screen four wells were treated with 3 µM of 10-E-09 and four wells were treated with 10 µM of 10-E-09. In the CDKN2A screen, DAC (1 µM) was applied to four wells and TSA (100 nM) to four other wells. The rest of the plate was used for the treatment with screened compounds at approximated concentration of 5 µM. In the IRF4 screen 10 µl of cell suspension were transferred into PCR plates right after drug administration and grown in the PCR plate for 6 h at 37°C in the incubator for later heating before use of 1 µl for DC-PCR while the remaining 190 µl cells were kept in the incubator for confirmatory tests on cells treated with hits. In the CDKN2A screen, cells were kept in 96 well tissue culture plates for 3 days before transfer of 1 µl into PCR plates for dilution with PCR mastermix and subsequent PCR. Remaining cells (199 µl) were kept for confirmatory assays on hit-treated cells.

In the IRF4 screen, after 6 hours of incubation the PCR plate with 10 µl cell suspension per well was subjected to a short heat shock of 80°C for 60 sec. at maximum ramp speed of the PCR cycler (Eppendorf, Mastercycler pro-S). 1 µl of heated cell suspension was then transferred into another PCR plate filled with room temperature PCR mix supplemented with 1x protease inhibitor and 1 µM ethidium bromide. After 15-25 minutes of incubation at room temperature PCR amplification was started and run for 40 cycles. PCR conditions: IRF4, 94°C, 5 min. → (94°C, 30 sec; 58°C, 30 sec; 72°C, 30 sec)x40 → 72°C, 1 min. → 4°C, hold. In the CDKN2A screen, after 72 h of incubation 1 µl of the cell suspension kept at 37°C was transferred into PCR plates filled with room temperature PCR mix including 1x protease inhibitor and 1 µM ethidium bromide. After 15–25 minutes of incubation at room temperature PCR amplification was started and run for 40 cycles. PCR conditions: CDKN2A, (94°C, 30 sec; 64°C, 30 sec; 72°C, 30 sec)x40 → 72°C, 2 min. → 4°C, hold.

Plates were analyzed visually and by densitometry under UV-light transillumination or on Wallac 1420 Workstation plate reader (Table S2). Remaining hit treated cells were harvested 3 h later for CDKN2A RT-PCR and 18 h later for IRF4 immunoblots in respective screens as a secondary assays.

### RT-PCR, Immunoblots and Chromatin Immunoprecipitation

RNA was isolated using RNeasy mini kit (Qiagen). cDNA synthesis was performed on iScript cDNA synthesis kit (Bio-Rad). DNA oligonucleotides were synthesized by IDT technology. Primer sequences and PCR conditions are in Table S3. Antibodies for Western Blot or CHiP assay: mouse, monoclonal to p16 (50.1) SC-9968 (Santa Cruz Biotechnology); Rabbit to IRF4, #4964S (Cell Signaling technology); Rabbit to NFκB1 p105/p50, #3035P (Cell Signaling technology); β-Actin, #4967L (Cell Signaling technology); mouse monoclonal to Histone H3 acetyl K9, AH3-120 (Abcam). All ChIP assays were done using the EZ ChIP Chromatin Immunoprecipitation Kit (Millipore) according to manufacturer’s instructions. Mouse antibodies to RNApol II and control mouse IgG were provided with the ChIP Kit.

### CD34 Purification of Cord Blood and Flow Cytometry

Magnetic beads (CD34 MicroBead Kit, Miltenyi Biotec Inc.) were used to enrich cord blood mononuclear cells obtained by ficoll gradient centrifugation according to the manufacturer’s instructions. After purification they were put in culture as outlined above with incubation at least over night before experiments were performed.

Flow cytometry was performed on FC500 with CXP Version 2.2 software (Beckman Coulter) after staining with Anti-CD34 (Clone AC136, Miltenyi Biotec). Co-staining with 7-AAD (7-amino actinomycin, BD biosciences), a DNA intercalating dye that does not pass intact cell membranes, was used to detect damaged and dead cells. 10,000 cells were analyzed per run and the gates were set on unstained controls.

### Global Gene Expression Analysis

Illumina Ht-12 arrays were used according to the manufactures instructions on Illumina Beadstation and expression analyzed using GenomeStudio version 2011.1.

## Results

### Direct Chromatin PCR (DC-PCR) Development

**Figure 1:**
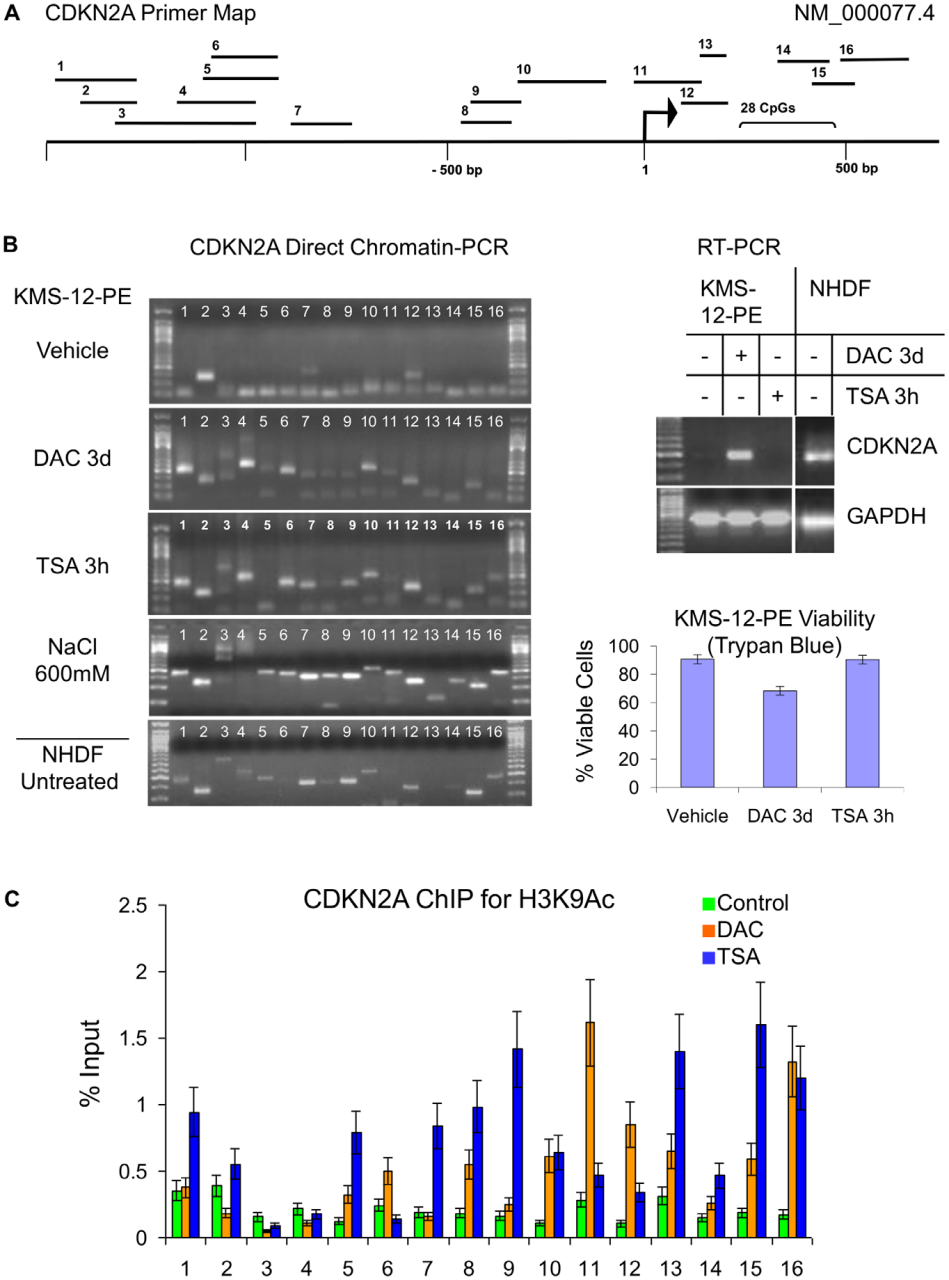
Direct chromatin PCR for CDKN2A gene. CDKN2A primer locations for DC-PCR are shown on a map of its 5′ region. An arrow indicates the transcription start site for transcript NM_000077.4 (**A**). Analysis of CDKN2A transcription by RT-PCR (Right upper panel) and chromatin structure by DC-PCR (Left panel) in control (vehicle treated) KMS-12-PE cells, after treatment with 1 µM decitabine (DAC) or 100 nM trichostatin (TSA) and in normal fibroblasts (**B**). KMS-12-PE multiple myeloma cells do not express CDKN2A (Right upper panel) and when untreated cells are subjected to DC-PCR, most CDKN2A primers do not yield products (Left panel). After treatment with DAC, CDKN2A mRNA expression is re-activated (Right upper panel) and DC-PCR yields multiple amplicons (Left panel). Short-term treatment with the deacetylase inhibitor TSA, which did not affect viability of KMS-12-PE cells (Right lower panel) also made chromatin accessible for DC-PCR amplification (Left panel) but did not induce CDKN2A mRNA expression (Right upper panel). Exposure of KMS-12-PE cells to high salt concentrations (600 mM NaCl for 20 min) allowed DC-PCR amplification of almost all CDKN2A sites. DC-PCR also detects the open chromatin structure of CDKN2A expressing normal human dermal fibroblasts (NHDF) (Left and Upper right panel, respectively). Correlation of DC-PCR results with Chromatin Immunoprecipitation (**C**). Chromatin immunoprecipitation assay of the CDKN2A regulatory region with antibodies against acetylated lysine 9 histone 3 (H3K9Ac) in control, vehicle treated cells and cells treated with DAC or TSA as under (B). Purified DNA was analyzed by real-time PCR using primers for the CDKN2A regulatory region shown on the map in Figure 1A. Error bars represent standard deviations of the mean of duplicates. All results are representative of at least three independent experiments.

**Figure 2:**
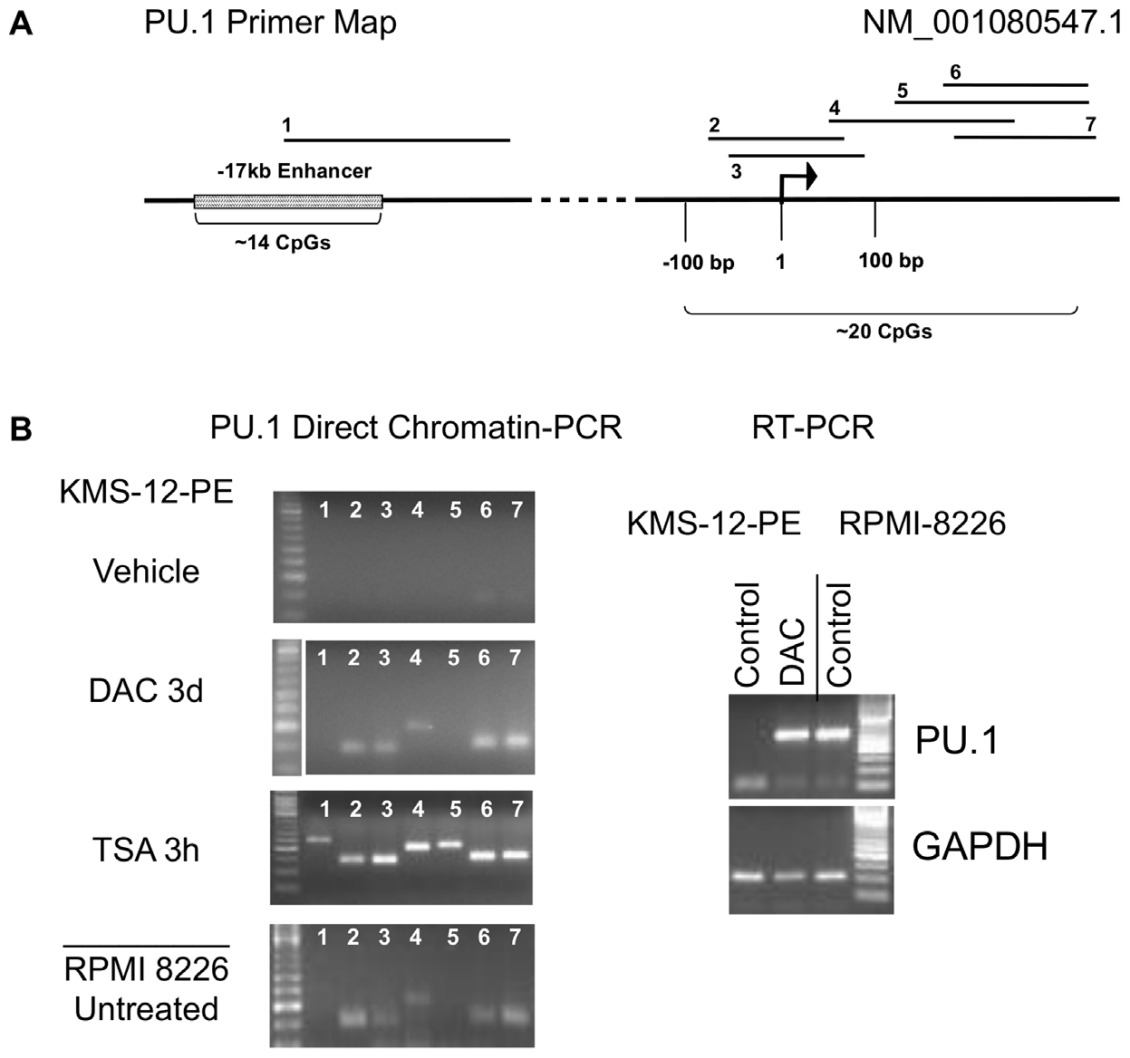
Direct chromatin PCR for PU. 1 gene. Primer locations for DC-PCR are shown on a map of the PU.1 5′ region (**A**). An arrow indicates the transcription start site for transcript NM_001080547.1. Analysis of PU.1 transcription by RT-PCR (Right panel) and chromatin structure by DC-PCR (Left panel) in control (vehicle treated) KMS-12-PE cells, after treatment with DAC (as described under Figure 1) and in PU.1 positive cells (**B**). The RPMI-8226 cell line expresses PU.1 while KMS-12-PE cells have PU.1 silenced but it can be re-activated by DAC treatment (Right panel). DC-PCR correlates with RT-PCR results: In expressing cells, whether at baseline or after treatment with DAC, most 5′ PU.1 sequences can be amplified while untreated KMS-12-PE cells yielded no products (Left panel). Brief TSA treatment of KMS-12-PE cells as under Figure 1, also opened PU.1 chromatin (Left panel). Displayed results are representative of three independent experiments.

During experiments aimed at streamlining site-specific chromatin analysis for application in drug discovery we included PCR of unprocessed cells as one of the controls using cells grown in RMPI 1640 media diluted at 1∶25 in standard hypotonic PCR buffer supplemented with protease inhibitors. To our surprise only three of 23 tested sequences 5′ of CDKN2A and PU.1 were amplifiable in cells that did not express respective genes (Figures 1 and 2) despite cyclic heating to temperatures above the melting point reported for extracted condensed chromatin (84–86°C) [10], [11]. However, after treatment with the DNA demethylating agent decitabine at 1 µM for 3 days, leading to mRNA reactivation in KMS-12-PE myeloma cells, similar DC-PCR results were obtained as in cells expressing CDKN2A (Normal Human Dermal Fibroblasts, NHDF) or PU.1 (RPMI 8226 myeloma cells) at baseline (Figures 1B and 2B). NHDF were removed from plates by tripsinization prior to DC-PCR. Trypsinization at 37°C for up to 5 min followed by addition of media did not alter chromatin accessibility for CDKN2A or c-MYC primers compared to scraping which both preserved >90% viability (data not shown). The epigenetic activity of decitabine is dependent on cell divison and can only be seen after several days of treatment, which is associated with a decrease in viability from around 90% to around 70% (Figure 1B) even at 10x lower doses (data not shown). To assess whether this might underlie chromatin accessibility we tested over 300 small molecules that similarly reduced viability and found CDKN2A chromatin amplification comparable to decitabine only in 12 (data not shown). Brief treatment with the histone class I and II deacetylase inhibitor trichostatin (TSA) at 100 nM for 3 h, which did not affect viability of KMS-12-PE cells (Figure 1B) resulted in amplification of 18 of 23 sequences 5′ of CDKN2A and PU.1 (Figures 1B and 2B) and increased histone acetylation at H3K9 in a similar way as decitabine confirming that DC-PCR informs about chromatin state (Figure 1C). Transcription was not induced after 3h of TSA treatment (Figure 1B) and even longer TSA treatment did not reactivate CDN2A in KMS-12-PE cells (data not shown), an observation reported in other cells with completely silenced CDKN2A [12] suggesting that DNA demethylation is required for CDKN2A mRNA reactivation in these cells. Treatment of KMS-12-PE cells with salt concentrations (600 mM NaCl for 20 min) known to solubilize chromatin of nuclear extracts [6] made 14 of 16 investigated sequences 5′ of CDKN2A accessible for PCR primers, further supporting use of unprocessed cells for chromatin analysis (Figure 1B).

**Figure 3:**
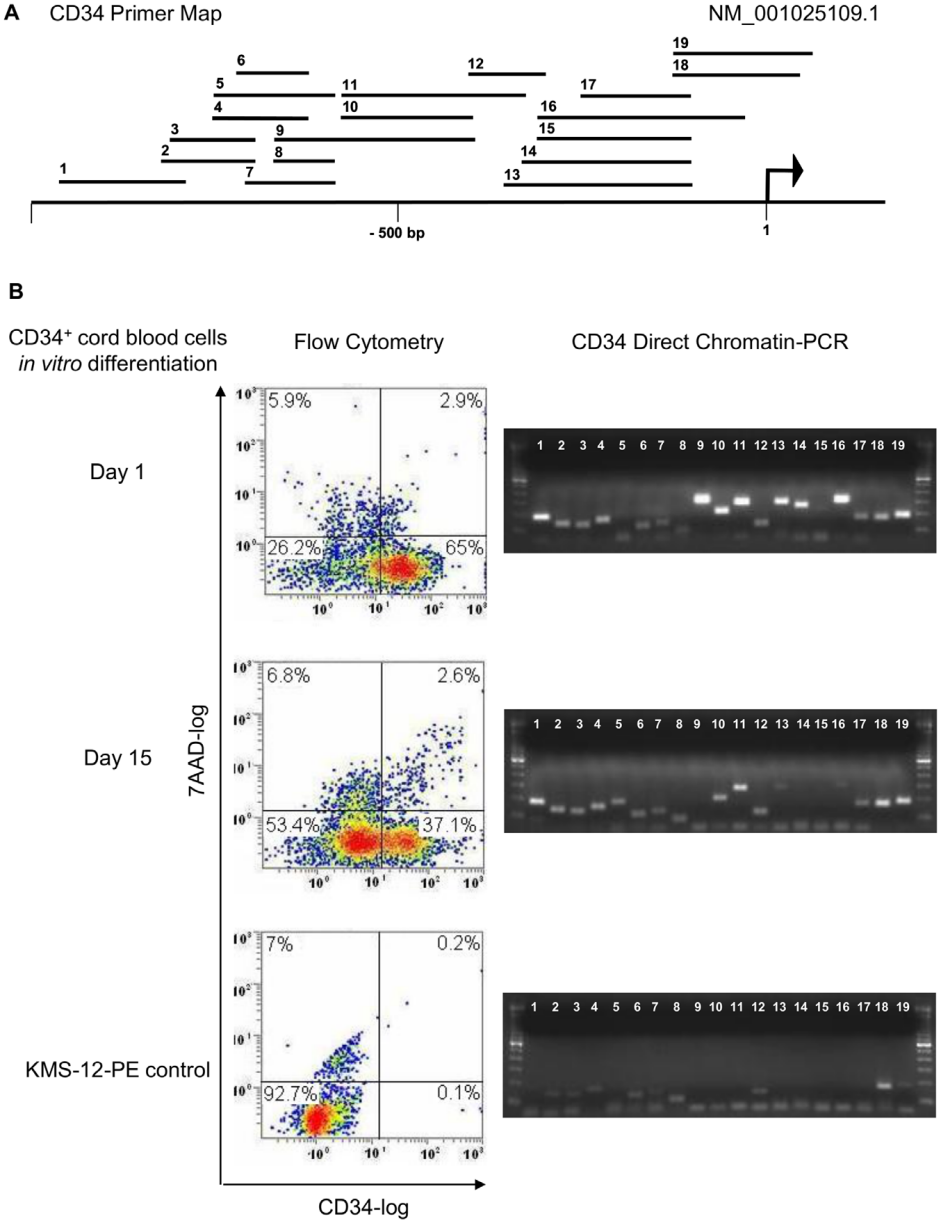
Direct chromatin PCR for CD34 gene: detection of epigenetic changes during cellular differentiation *in vitro*. Primer locations for DC-PCR are shown on a map of the CD34 5′ region (**A**). An arrow indicates the transcription start site for transcript NM_001025109.1. Flow cytometry (Left panel) and DC-PCR analysis (Right panel) of cord blood CD34+ cells as they undergo cytokine mediated differentiation *in vitro* (**B**). One day after CD34 magnetic bead purification about 65% of cells expressed CD34 as determined by flow cytometry (Left panel). At day 15 this fraction had decreased to 37.1%, while cells remained healthy with less than 10% showing evidence for loss of membrane integrity as assessed by 7-AAD co-staining (Left panel). CD34 DC-PCR (Right panel) identified decreased accessibility of sequences within about 500 bp 5′ of the CD34 transcription start site on day 15 compared to day 1 and of 11 tested sequences in this region (primers 9–19) only 3 yielded (faint) DC-PCR products in CD34 negative (Left panel) KMS-12-PE cells (Right panel). Results are representative of three independent experiments.

To investigate whether DC-PCR yields consistent results in settings other than reactivation of tumor suppressor genes by epigenetic agents, which directly act on chromatin, we studied CD34 positive cord blood cells that are known to grow and differentiate *in vitro*, gradually loosing CD34 expression, under the influence of cytokines [13]. After CD34 magnetic bead purification cells were grown in cytokine containing culture. One day after purification 65% of cells expressed CD34 as determined by flow cytometry; at day 15 this fraction had decreased by almost half to 37.1%, while cells remained healthy with less than 10% showing evidence for loss of membrane integrity as assessed by 7-AAD co staining. CD34 DC-PCR showed decreased accessibility of sequences within about 500 bp 5′ of the CD34 transcription start site on day 15 compared to day 1 and of 11 tested sequences spanning this region (primers 9–19) only 3 yielded DC-PCR products in CD34 negative KMS-12-PE cells which were used as control (Figure 3). Thus, DC-PCR can also detect epigenetic changes during differentiation.

**Figure 4:**
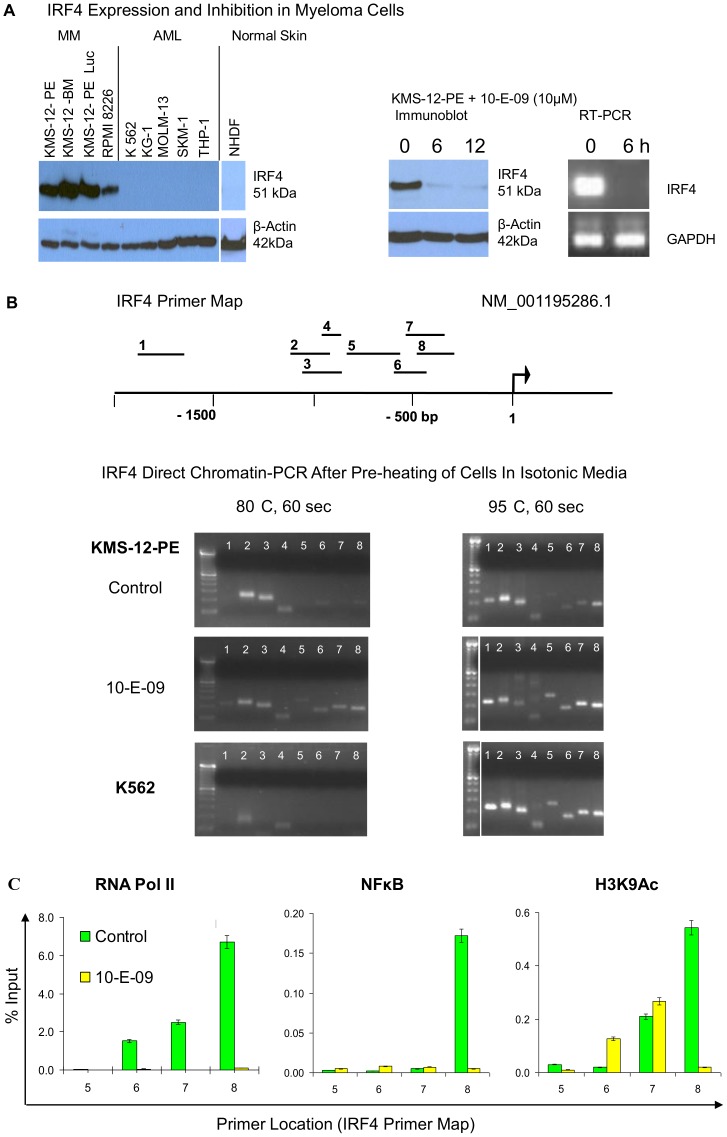
Direct chromatin PCR for IRF4 gene. IRF4 Expression and Inhibition in Myeloma Cells. Myeloma cells express high levels of IRF4, while leukemia cells or normal fibroblasts (NHDF) have IRF4 silenced. Treatment with 10-E-09 suppresses IRF4 mRNA and protein expression (upper right panel) (**A**). Primer locations for DC-PCR are shown on a map of IRF4 5′ region (**B**). An arrow indicates the transcription start site for transcript NM_001195286.1. After pre-heating of cells to 80°C for 60 seconds in isotonic media, sites 5′ to the IRF4 transcription start site became more accessible to DC-PCR amplification in IRF4 expressing KMS-12-PE cells than in IRF4 negative K562 cells (Left panel). The sites closest to the transcription start site only opened after treatment with the IRF4 inhibitor 10-E-09 at 10 µM for 6 h (Left panel). After pre-heating of cells in isotonic media to 95°C for 60 seconds multiple IRF4 chromatin sites could be amplified in all cells (Right panel). Displacement of RNA polymerase II and NFkB from the IRF4 promoter and changes in the level of H3K9Ac after treatment of KMS-12-PE myeloma cells with the IRF4 inhibitor 10-E-09 investigated by ChIP (**C**). The chromatin immunoprecipitation assay used antibodies against RNA polymerase II, NFkB and acetylated lysine 9 histone 3 (H3K9Ac) in vehicle (DMSO) treated cells and in cells treated with 10 M of 10-E-09 for 6 hours. Purified DNA was analyzed by real-time PCR using primers #5, 6, 7 and 8 of the IRF4 promoter. Error bars represent standard error of the means of duplicates. All results are representative of at least three independent experiments.

For drug screening purposes we were most interested in analyzing the myeloma survival factor IRF4 [14] since our translational laboratory focuses on this disease. Wondering why temperatures as high >90°C would not open repressed chromatin, we were at the same time unsuccessful at amplifying the regulatory region of IRF4 even in strongly expressing myeloma cell lines (Figure 4A) using a 5 min hot start at 95°C and primers that gave strong and clean products when applied to isolated DNA (data not shown). We hypothesized that the low salt concentration of standard PCR buffers (50 mM KCl, 10 mM Tris-HCl, 1.5 mM MgCl_2_, pH 8.3) was protecting chromatin from heat solubilization preventing primer access even in more organized active regions. Therefore we tested brief exposure of cells to increasing temperatures in isotonic tissue culture media before dilution into PCR buffer, which can be accomplished without additional pipetting steps in high-throughput drug screening applications. Indeed, when cells were pre-heated to 80°C for 60 sec in this isotonic environment containing 103.45 mM NaCl and other less abundant salts (see Materials and Methods for details), differentiation between expressing and non-expressing cells by the number of amplifiable 5′ sequences was possible but the transcription factor binding region remained inaccessible even in highly expressing myeloma cells (Figure 4A, lower left panel). Pre-heating of cells to 95°C for 60 sec in isotonic tissue culture media allowed amplification of all IRF4 regulatory sequences in expressing and IRF4 silenced cells (Figure 4A, lower right panel). This confirmed the chromatin stabilizing effect of standard PCR buffer, where even longer exposure to the same temperature did not open cellular chromatin. The relative resistance to heat solubilization of the transcription factor binding region in myeloma cells suggested that strong interactions with transcriptional complexes prevented primer access. We had previously identified a transcriptional inhibitor of IRF4 (10-E-09) that almost completely abrogates IRF4 mRNA and protein expression at 6 h (Figure 4A, upper right panel) about 12 h before viability is affected (Vi-CELL based Coulter counting with trypan blue exclusion). DC-PCR of mildly pre-heated (80°C, 60 sec) KMS-12-PE cells 6 h after treatment with 10-E-09 yielded PCR products for the transcription factor binding region, and ChIP confirmed dissociation of RNA polymerase II and NFκB with reduction of the histone activation mark H3K9 acetylation in the sequence closest to the transcription start site (Figure 4B).

**Figure 5:**
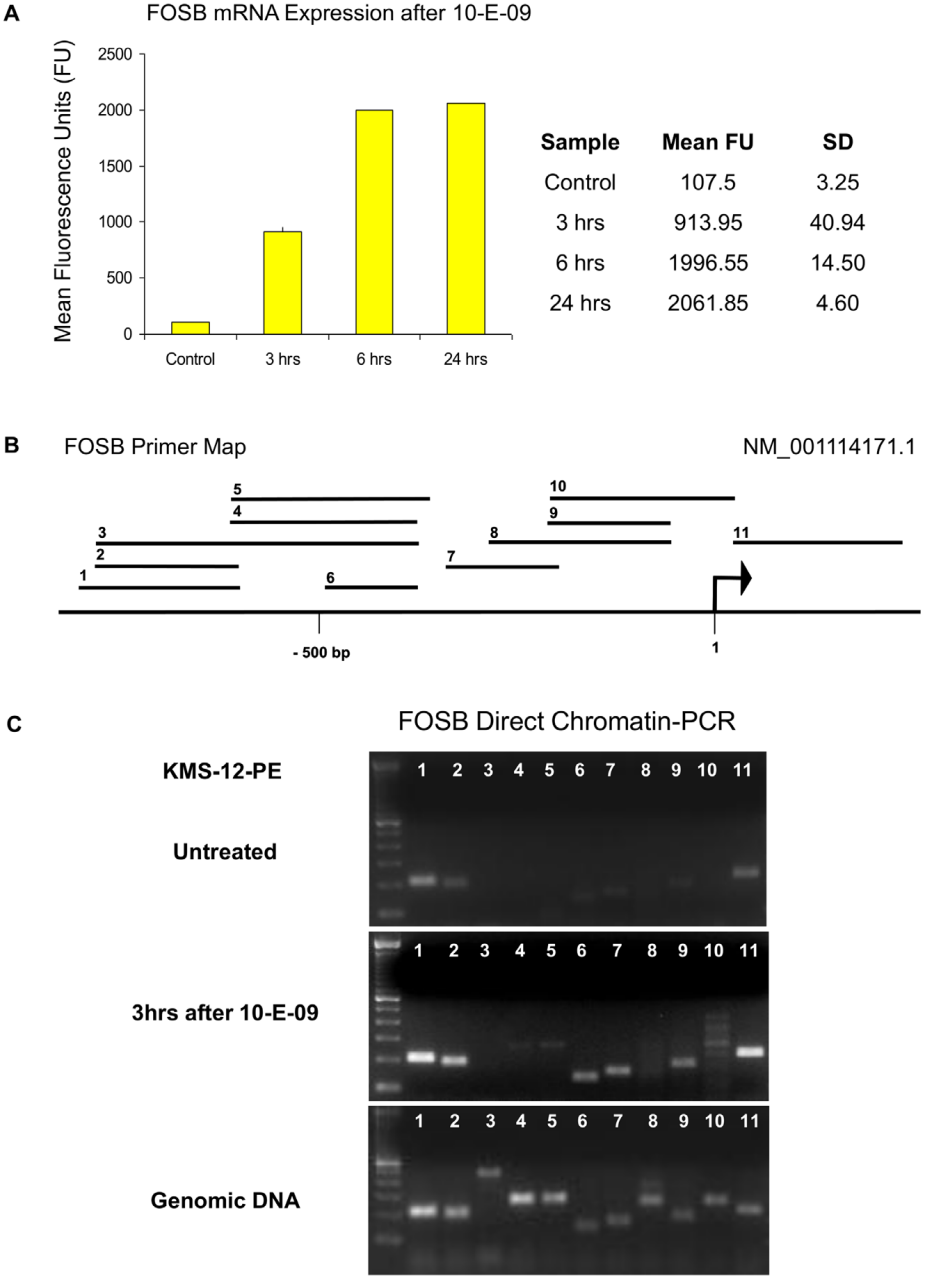
Direct chromatin PCR for FOSB gene. FOSB mRNA Expression after 10-E-09 In KMS-12-PE detected and analyzed on HT-12 Array™ (**A**). Cells were treated with 10-E-09 at 10 µM for indicated times and arrays were run in duplicates. Displayed are mean fluorescence units (Left panel) and standard deviations of duplicates (Right panel). Primer locations for DC-PCR are shown on a map of the FOSB 5′ region (**B**). An arrow indicates transcription start site for transcript NM_001114171.1. DC-PCR performed for FOSB on KMS-12-PE cells (**C**): untreated and treated with 10-E-09. Active chromatin of FOSB can be detected by DC-PCR 3 h after treatment with 10-E-09. Most of the 11 tested sequences allowed primer access whereas chromatin of untreated cells remained almost completely closed. Genomic DNA used as template demonstrated amplification of all FOSB sites. Displayed DC-PCR results are representative of three independent experiments.

To further confirm generalizability of results we selected a gene from an unbiased global gene expression experiment that was reactivated within 3 h after treatment of KMS-12-PE cells with 10-E-09, the leucine zipper protein FOSB (Figure 5A). This gene, like IRF4 required pre-heating of cells in isotonic media to 80°C for 60 sec before its chromatin became accessible but 3 h after treatment with 10-E-09 most of the 11 tested sequences allowed primer access whereas chromatin of untreated cells remained almost completely closed (Figure 5C). Another interesting observation was made when the area around the FUSE element, involved in transcription control of c-MYC [15] was analyzed by DC-PCR. Treatment of KMS-12-PE cells with decitabine (1 µM) for 3 days resulted in similar chromatin amplification by DC-PCR of an area 5′ of the FUSE element as did growth arrest of normal human dermal fibroblasts who had reached confluence (Figure S1) but still had high viability (>90% trypan blue negative). Thus epigenetic changes of growth controlling genes can also be detected by DC-PCR.

In summary, investigation of 65 sequences by DC-PCR suggests that the technique can be used to differentiate chromatin states in cells directly or for more organized regions, after brief pre-heating in isotonic media since hypotonic PCR buffers stabilize chromatin during thermal cycling. While the majority of analyzed sequences were more accessible in expressing cells, the IRF4 example illustrates that binding of transcriptional complexes can also prevent primer access.

### Adaptation to Small Molecule Screening

**Figure 6:**
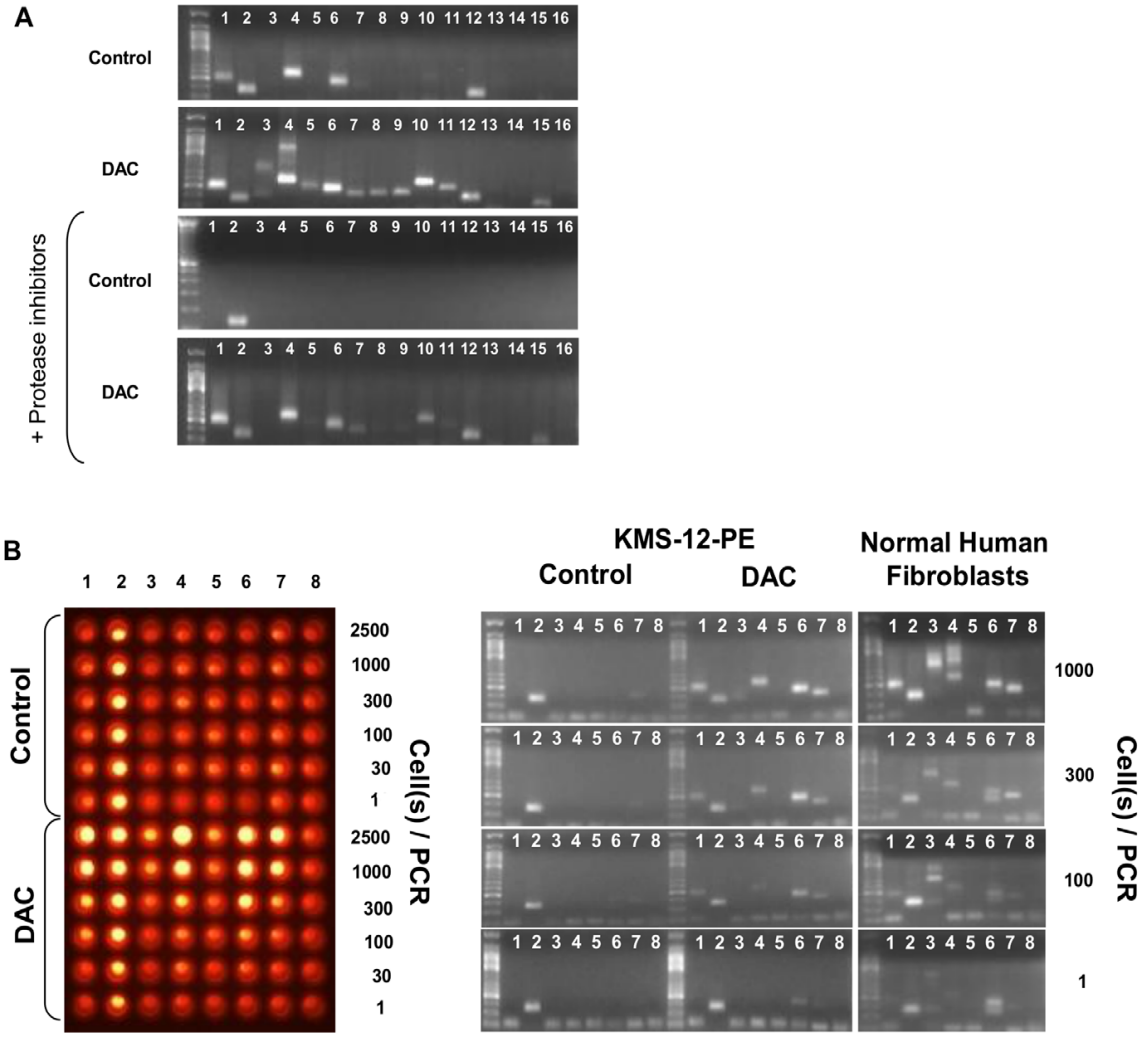
Optimization of DC-PCR for drug screening. Effect of cytoplasmic enzymes on DC-PCR (**A**). KMS-12-PE cells were diluted at 1∶25 in hypotonic PCR buffer with or without added protease inhibitors and incubated for 20 min before PCR was run to simulate the time required for pipetting during a small molecule screen. Inclusion of protease inhibitors in the PCR mastermix decreased the number of amplifiable sites in DAC treated and untreated KMS-12-PE cells suggesting that digestion of DNA binding proteins can occur in PCR buffer although epigenetic differences between samples are maintained in the studied example. Detection of DC-PCR product via UV transillumination of PCR plates and sensitivity compared to gel electrophoresis (**B**). Ethidium bromide was added to the PCR mastermix before serially diluted cells were submitted to DC-PCR: untreated, DAC treated or fibroblasts. After completion of thermal cycling samples were first analyzed under UV light (Left panel), then via agarose-gel electrophoresis (Right panel). Using gel-based detection, the level of detection for epigenetic differences of CDKN2A was lower, down to about 1 cell for site #6 compared to about 300 cells with direct UV transillumination. The primer numbers correspond to sequences on the map described in Figure 1A. Three independent experiments yielded the same detection levels. Correlation between direct UV transillumination and gel electrophoresis was seen in all experiments (hundreds).

Before proceeding to high throughput use of DC-PCR we assessed whether pipetting time could influence results and determined the minimal amount of cells required for a positive readout while streamlining the method to 1-2 pipetting steps. Pipetting time would lead to incubation of cells in hypotonic PCR buffer which was expected to affect membrane integrity and possibly destabilize chromatin via enzymatic digestion. We focused on CDKN2A since its DNA-protein bonds appeared relatively weak. When incubation of cells in PCR buffer with or without protease inhibitors was compared, the number of amplifiable sequences indicated chromatin digestion took place during incubation but differentiation between baseline and decitabine treatment was possible with or without added protease inhibitors (Figure 6A). To avoid variation of results based on small changes in incubation time, we performed all other DC-PCR experiments shown in this manuscript in the presence of protease inhibitors. Use of commercially available real-time PCR buffers non-selectively opened cellular chromatin, which may be due to high detergent concentrations but the following allowed use of conventional DC-PCR for drug screening: In order to limit pipetting steps to one and define the minimum number of cells for a positive readout, we tested whether addition of ethidium bromide to PCR mastermix would yield comparable results on gel electrophoresis as with gel staining and performed serial dilution of cells comparing gel detection and detection of product by UV transillumination of PCR plates. Ethidium bromide addition to PCR mastermix did not affect gel electrophoresis results but the detection limit was lower by gel electrophoresis (about 1 cell) than by transillumination of PCR multiwell plates (around 300 cells, Figure 6B and Table S1). Since DC-PCR is run on 1 µl cell suspension, the final cell concentration after small molecule library treatment should therefore be at least around 30,000 per 100 µl if transillumination of PCR plates is used and could be as low as about 100 per 100 µl for gel based detection. Only 1 µl of cell suspension is needed for DC-PCR, remaining cells can be used for confirmatory tests or secondary screens. Since results of DC-PCR are available within 3 h, for most applications cells can be kept in the incubator while awaiting results, alternatively they can be frozen.

**Figure 7:**
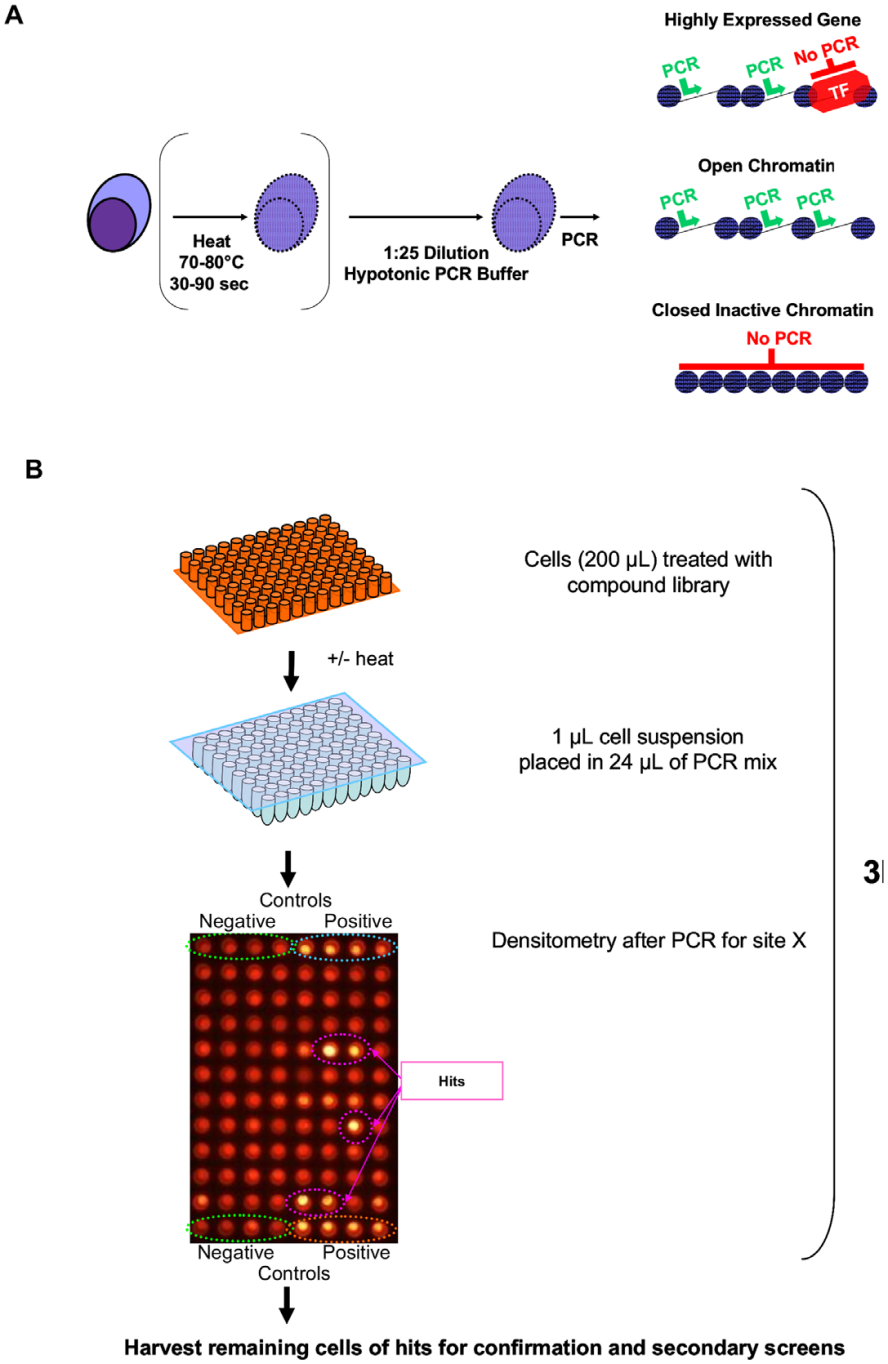
Principles of DC-PCR drug screens. Cartoon outlining the general procedure of DC-PCR and possible results that depend on the interrogated region and utilized cells (**A**). General outline of a CS-PCR screen for molecules that make the interrogated site more soluble (**B**). Only a fraction of cells (1–10 µl) is required to run the screen. Remaining cells can be used for confirmatory tests or secondary screens. If heat is used and confirmatory tests on hits are planned, an additional pipetting step before heating is required.

Figure 7B and Table S2 shows the general scheme of a DC-PCR small molecule screen and our understanding of the method based on analysis of over 60 sequences in the 5′ regions of 6 genes (Figure 7A).

### Direct Amplification with Phi29 Polymerase

**Figure 8:**
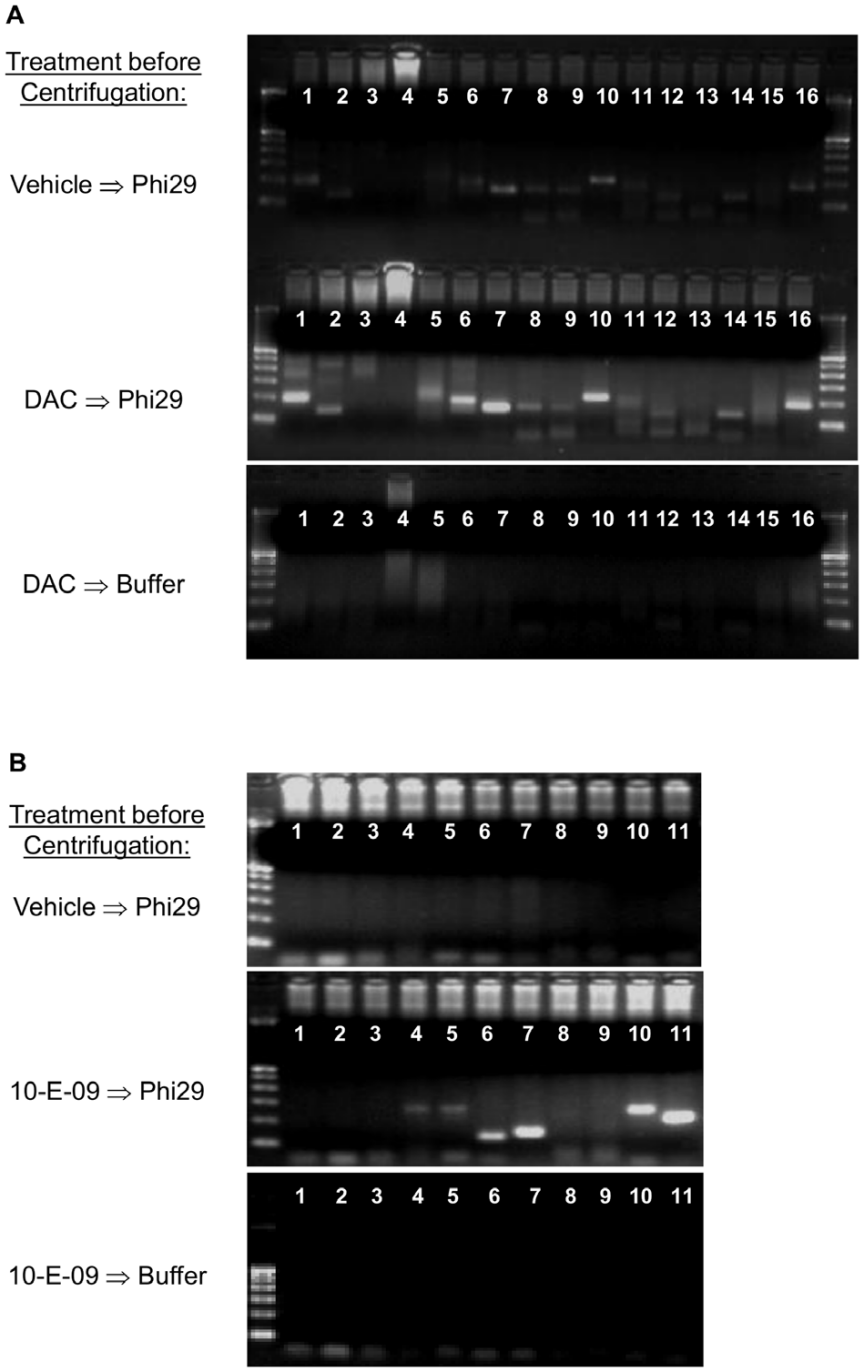
Direct Amplification of open chromatin with phi29 polymerase: Translation of DC-PCR to genome-wide chromatin analysis. CDKN2A PCR on KMS-12-PE supernatant after direct genome-wide phi29 amplification (**A**). Untreated KMS-12-PE cells or cells treated with DAC (1 µM for 3 d) were placed directly in phi29 reaction mix for 4.5 hrs at 30°C. After that, the reaction was centrifuged to pellet cell carcasses. Only the upper 50% of the supernatant was used for subsequent amplification of CDKN2A sites by conventional PCR. Supernatant from DAC treated cells yielded more CDKN2A products than vehicle treated cells. When DAC treated cells were incubated in reaction buffer without Phi29 polymerase no CDKN2A products could be amplified from supernatants by taq polymerase during conventional PCR. FOSB PCR on KMS-12-PE supernatants obtained from untreated cells or cells treated with 10-E-09 (10 µM for 6 h) after direct genome-wide phi29 amplification (**B**). The same procedure as above yielded FOSB PCR products from supernatants of 10-E-09 treated KMS-12-PE cells amplified by phi29 while vehicle treated cells or supernatants from 10-E-09 treated cells incubated in reaction buffer without phi29 yielded no FOSB regulatory region amplicons. Results are representative of three independent experiments.

To assess whether translation into a genome-wide chromatin analysis is feasible, we treated KMS-12-PE cells as before gene specific DC-PCR for CDKN2A (decitabine 1 µM for 3 days or vehicle) and FOSB (10-E-09 3 µM for 6 h or vehicle followed by heating to 80°C for 60 sec in isotonic media) before amplification with phi29 polymerase at 37°C for 4.5 h. Reactions were subsequently centrifuged and only the upper half of the supernatant was used for CDKN2A and FOSB PCR using primers as for gene specific DC-PCR. Supernatant of decitabine and 10-E-09 treated cells exposed only to buffer without phi29 polymerase served as controls. Similar differences between treated and untreated cells were observed (Figure 8A, B), suggesting that phi29 can be used for genome-wide analysis of chromatin states.

### Small Molecule Screens

Initially we performed a 5120 small molecule screen for reactivators of CDKN2A using primer sequences flanking site 15 (Figure 1). TSA and decitabine served as positive controls, untreated and vehicle treated cells as negative controls. Small molecules were applied at 5 µM and cells were then incubated for 3 days before DC-PCR was performed. At that time, 96 well tissue culture plates were also inspected for lack of media color change to screen for cytotoxic compounds. 148 compounds enabled amplification by DC-PCR as determined by densitometry signals at least as high as the average of decitabine and TSA treated wells and 315 prevented media color change. Only 12 did both. Remaining cells of CDKN2A hits were harvested for RT-PCR and of 15 CDKN2A mRNA reactivating hits, 14 could be confirmed in at least 3 independent experiments (Figure S2). The relatively high rate of CDKN2A chromatin reactivation compared to mRNA re-expression was not surprising given reports for the need of DNA demethylation to reactivate completely silenced CDKN2A [12]. None of the CDKN2A mRNA reactivating hits were nucleoside analogs but 8 inhibited DNA methyltransferase I (data not shown). Untreated cells and cells treated with vehicle maintained viability of 85-95% while trypan blue positive cells of wells without media color change ranged between 8 and 75%.

**Figure 9:**
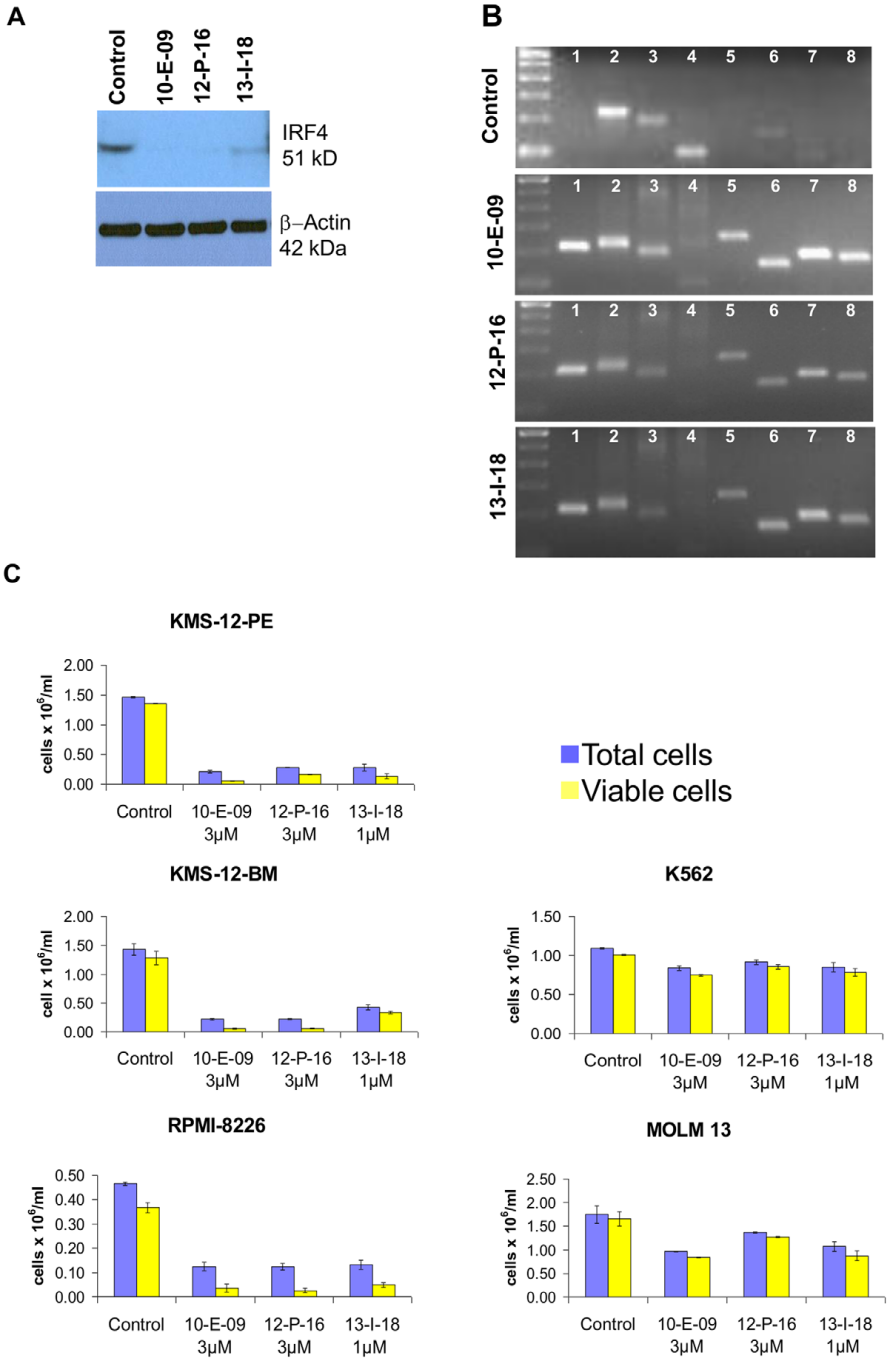
Inhibitors of IRF4 expression identified by DC-PCR. Reduction of IRF4 protein expression by hits in myeloma cells compared to 10-E-09. Immuno-blot for IRF4 was performed after treatment with vehicle (DMSO) or with drug-candidate inhibitors at 3 µM for 24 hours (**A**). β-Actin was used as loading control (lower panel). Similar results were obtained in three independent experiments. Confirmatory gel electrophoresis of DC-PCR after treatment of KMS-12-PE cells with drug candidates (IRF4 inhibitors) at 3 µM for 6 hours (**B**). The solubilization of sites 7 and 8 indicates that IRF4 transcriptional complexes are displaced by hits suggesting at least a common final path of all three IRF4 expression inhibitors. Results were confirmed in at least three independent experiments per drug candidate. Treatment with hits suppresses growth and survival of IRF4 expressing multiple myeloma cells (Left panels) more than IRF4 negative AML cell lines (Right panels) (**C**). Cells were treated with IRF4 inhibitors at indicated concentrations for five days before coulter counter with trypan blue exclusion was used to assess total and viable cell counts. Error bars represent standard deviations of least two independently treated wells. Similar results were obtained in at least one independent repeat per cell line.

Due to potential therapeutic use in our patient population we were most interested in identifying small molecule inhibitors of the multiple myeloma survival factor IRF4 [14]. Multiple myeloma cells have high constant expression of this gene which is never expressed in the majority of cells except in myeloid and lymphoid cells, where activation occurs during certain stages of maturation and in response to external stimuli or viral infections [5]. Using 10-E-09 as control we assessed 124 small molecules pre-selected from a library of 5120 diverse chemical compounds by their selective growth inhibitory effect on KMS-12-PE myeloma cells compared to IRF4 negative fibroblasts. In the DC-PCR screen KMS-12-PE cells were treated with small molecules at 5 µM for 6 h before DC-PCR using IRF4 primer #8 (Figures 4A and 9B) was performed. Of 8 hits on UV transillumination and gel electrophoresis, two yielded similarly profound IRF4 protein suppression as 10-E-09 (Figure 9A). Their growth inhibitory actions on myeloma cells compared to IRF4 negative leukemia cells appeared equally selective as with 10-E-09 (Figure 9C). Medicinal chemistry optimization of these candidates is on-going. Results of this pilot screen demonstrate that whole cell DC-PCR can be used to identify small molecules that affect chromatin solubility at sites of interest.

## Discussion

We show here that site specific chromatin solubility can be analyzed by submitting cells to PCR since standard hypotonic PCR buffers prevent universal chromatin solubilization during thermal cycling while less densely organized regions are amplified.

The chromatin stabilizing effect of hypotonic PCR buffer during repetitive heating can even prevent amplification of more organized active regions. As we show here for IRF4 and FOSB, a pre-heating step in isotonic media can allow differentiation between active and inactive chromatin (Figures 4 and 5). Since the melting point for inactive chromatin varies depending on the analyzed site, it is necessary to perform a temperature titration experiment with well characterized active and inactive controls before setting up a drug screening experiment. As demonstrated here for IRF4, and reported after salt fractionation of nuclei [6], DNA interactions with transcriptional complexes can be stronger than found in inactive chromatin (Figure 4A). Thus a lack of PCR product on DC-PCR can mean either repression of chromatin or strong expression, depending on the analyzed site. Of 65 analyzed sequences 5′ of six genes only 3 (4.6%) were shown to display strong heat-resistant DNA-protein interactions due to transcription factor binding, a fraction that correlates with results of salt treatment of nuclei from drosophila where such sites were found in the 5% of the genome that did not solubilize in 600 mM NaCl [6]. Much more commonly amplification by DC-PCR indicated epigenetic activity; in 5 genes (CDKN2A, PU.1, CD34, IRF4, FOSB) with epigenetically defined control cells amplification of 5′ sequences by DC-PCR was achieved in 47 of 61 tested sites for epigenetically active cells whereas only 20 were amplifiable in epigenetically inactive cells (Figures 1 and 2). DC-PCR results correlated with expression (Figures 1, 2, 3, 4, 5), ChIP (Figures 1 and 4B), epigenetic treatment (Figures 1 and 2), cytokine-mediated differentiation (Figure 3), and growth inhibition (Figures 1, 2, 4, 5 and 9B).

Commercially available real-time PCR buffers non-specifically opened chromatin but drug screening was made simple by inclusion of ethidium bromide in the PCR mastermix which allowed analysis for hits using UV transillumination of PCR plates in 1–2 pipetting step with a threshold for differentiation of epigenetic states of about 300 cells (Figure 6B). If a higher sensitivity is needed, capillary (Pixel, Qiagen) or ultra thin layer gel electrophoresis [16] may allow automation and should be able to distinguish chromatin states of single cells (Figure 6B). Addition of protease inhibitors to PCR mastermix prevents enzymatic chromatin digestion during incubation of cells in hypotonic PCR buffer at room temperature, before cycling conditions inactivate enzymes by denaturation (Figure 6A). While additional optimization for direct real-time chromatin PCR may augment utility for high-throughput screens,.even with ethidium bromide-based detection this is to the best of our knowledge the simplest method to investigate chromatin states in drug screens.

Our main goal was to develop a method for identification of small molecules that are able to activate or repress chromatin of genes of interest through any mechanism to allow drug development for relevant targets even if regulating pathways are not known. Most gene silencing pathways, many gene suppression mechanisms, and the majority of gene specific epigenetic activating signals are actually not known but might be discovered if respective chemical agents were available. Other described methods did not allow application to larger scale drug screens for site specific chromatin modifiers. Chromatin salt fractionation, which established that binding of large transcriptional complexes can make active chromatin regions salt insoluble, requires nuclear extraction and enzymatic digestion by micrococcal nuclease [6], increasing complexity and required considerably higher cell numbers therefore severely limiting its application to drug screening. Another disadvantage of nuclear extraction in the context of drug screening is that it involves the use of detergent at concentrations high enough to alter chromatin structure in a time and cell dependent fashion [17]. Other ways to examine chromatin are limited to nucleosome-free regions and also require multiple experimental steps including centrifugation and relatively high cell numbers [7], [8], [9]. One alternative to screen for chromatin and transcriptional modifiers is to use direct RT-PCR, but this does not necessarily yield hits acting on chromatin or even at the level of transcription and is substantially more expensive. Further, unless a complete lack of expression represents baseline or readout, cell death and small differences in cell numbers can lead to false calls. Cell death did not result in false positive results on DC-PCR when CDKN2A chromatin activation was investigated after treatment with cytotoxic agents but induction of cell death can lead to dissociation of transcription factors from the IRF4 promoter yielding false positive results when the assay is run 48 h after treatment with cytotoxic agents (data not shown). When screening for transcriptional suppressors that enable amplification of respective sites on DC-PCR, like performed here for IRF4 (Figures 4 and 7), an early drug incubation time point of 2–6 h can be used to limit the number of indirectly acting hits as well as false positives. The added half-life of RNA and lack of transcription specificity makes direct RT-PCR not only more expensive but also less informative technique when searching for gene expression modulators. Although similarly simple to run, vector-based screens that link luciferase expression to epigenetic modification of sequences of interest are not only more time consuming during the screen setup-phase and not readily applicable to any gene, but also suffer from cellular epigenetic responses to the vector itself. This can result in vector silencing and hits may not have the same effect on non-transfected cells, an experience we gained with a PU.1 responsive lentiviral vector (data not shown), that contributed to our desire to establish the presented method. A careful setup phase in well defined positive and negative controls to establish whether pre-heating of cells is required and which of about 10–20 primer pairs covering the respective regulatory region differentiates best between desired outcomes is also necessary for successful use of DC-PCR but this can be completed in less than one week.

Using IRF4 expression inhibition as an endpoint for drug candidate identification we found agents that at least in preliminary screens appear to possess selective actions against multiple myeloma cells (Figure 9). Although we pre-selected agents for this pilot screen by growth inhibition on myeloma cells and lack of cytotoxicity in normal fibroblasts we expect large scale screens should yield similarly selective agents among hits. For genes with well defined roles in pathogenesis of any disease, DC-PCR provides a straightforward method for identification of modifiers.

The high sensitivity with a detection limit of 1–10 cells if gel electrophoresis is used as readout, and the fact that there is no cell loss from centrifugations make the procedure attractive for use in translational research, especially on small clinical samples. While this will require defining the effect of post-harvest processing of specimens on results, standardized processing should readily allow application to gene specific interrogation. In preliminary experiments using phi29 polymerase during PCR instead of gene specific primers we were able to distinguish epigenetically treated from untreated cells on subsequent PCR of amplified DNA for CDKN2A and FOSB regulatory regions (Figure 8). Thus, coupling the principles of DC-PCR with high fidelity methods for whole genome amplification, like phi 29 polymerase, and next generation sequencing may enrich available methods for comprehensive analysis of chromatin states but is beyond the scope of this initial method description.

Site-specific chromatin solubility can be analyzed with the herein described simple procedure which allows conclusions regarding chromatin activity and occupancy of specific regions with transcriptional complexes after 1–2 pipetting steps. Applied in drug screens (Figure 7, 9 and Figure S2) it can yield clinically meaningful agents and it offers promise for simplifying gene specific and genome-wide translational chromatin research.

## Supporting Information

Figure S1 **c-MYC Direct Chromatin-PCR (DC-PCR).** KMS-12-PE cells were treated with DAC at 1µM for three days before c-MYC DC-PCR with primers flanking the FUSE element was performed. Normal human fibroblasts in exponential growth phase or growth arrested by reaching confluence served as comparison. Similar amplification with primers spanning the region 5′ of the FUSE element was observed with DAC treatment and with growth inhibition by confluence.
(PDF)

Figure S2 **CDKN2A mRNA reactivating hits from a direct chromatin PCR (DC-PCR) screen.** KMS-12-PE cells (200 µl at 100,000 cells/ml) were treated with a small molecule library containing 5120 diverse chemical compounds at 5µM for three days before 1 µl cell suspension was used for DC-PCR, remaining cells were left in culture. Three hours later DC-PCR hits were known and hit-treated cells harvested for CDKN2A RT-PCR. Shown are fourteen non-nucleoside compounds which reactivated CDKN2A expression in at least three independent experiments. Vehicle treated cells served as negative control, DAC treatment at indicated concentrations for three days as positive control, and water during RT reaction and during PCR as RT-PCR control.
(PDF)

Table S1 CDKN2A DC-PCR plate densitometry of decreasing cell numers. The relative DNA concentration in a plate shown on Figure 6B after staining with ethidium bromide read and quantified on Wallac 1420 Workstation plate reader.
(XLS)

Table S2 Exemplary DC-PCR plate densitometry of small molecule screen. The relative DNA concentration in a plate shown on Figure 7B after staining with ethidium bromide read and quantified on Wallac 1420 Workstation plate reader.
(XLS)

Table S3 Primer sequences, location and predicted sizes of PCR fragments.
(XLS)
