# Supplementary material for: Direct Chromatin PCR (DC-PCR): Hypotonic Conditions Allow Differentiation of Chromatin States during Thermal Cycling
Source: PLoS One. 2012 Sep 12;7(9):e44690. doi: 10.1371/journal.pone.0044690 (PMC3440349; doi:10.1371/journal.pone.0044690)

c-Myc Primer Map NM_002467.4

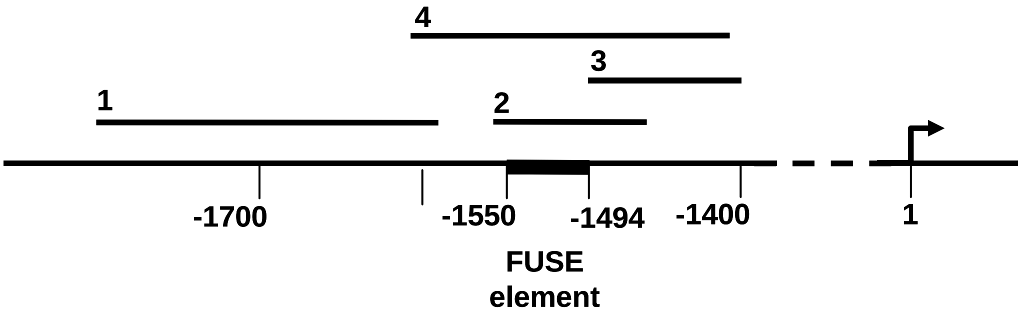

c-MYC Direct Chromatin-PCR

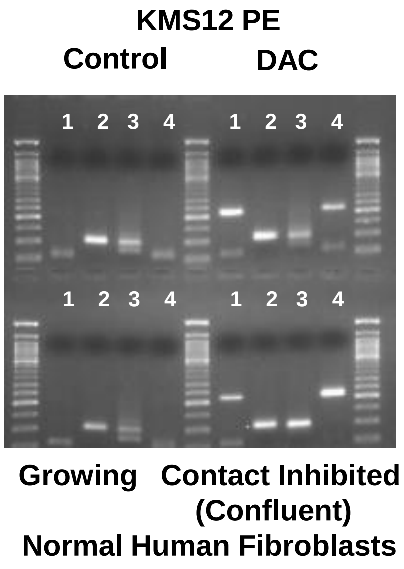

Supplement: Figure S1 — c-MYC Direct Chromatin-PCR (DC-PCR). KMS-12-PE cells were treated with DAC at 1µM for three days before c-MYC DC-PCR with primers flanking the FUSE element was performed. Normal human fibroblasts in exponential growth phase or growth arrested by reaching confluence served as comparison. Similar amplification with primers spanning the region 5′ of the FUSE element was observed with DAC treatment and with growth inhibition by confluence. (PDF) [file pone.0044690.s001.pdf]
