# Supplementary figures and images for: Direct Chromatin PCR (DC-PCR): Hypotonic Conditions Allow Differentiation of Chromatin States during Thermal Cycling
Source: PLoS One. 2012 Sep 12;7(9):e44690. doi: 10.1371/journal.pone.0044690 (PMC3440349; doi:10.1371/journal.pone.0044690)

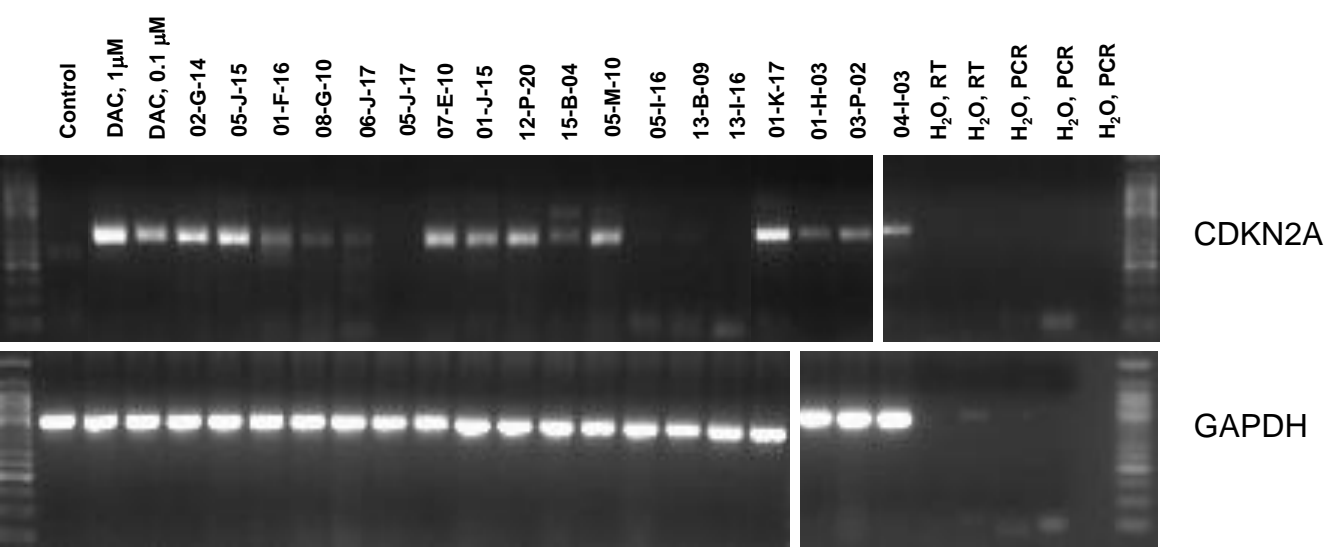

Supplement: Figure S2 — CDKN2A mRNA reactivating hits from a direct chromatin PCR (DC-PCR) screen. KMS-12-PE cells (200 µl at 100,000 cells/ml) were treated with a small molecule library containing 5120 diverse chemical compounds at 5µM for three days before 1 µl cell suspension was used for DC-PCR, remaining cells were left in culture. Three hours later DC-PCR hits were known and hit-treated cells harvested for CDKN2A RT-PCR. Shown are fourteen non-nucleoside compounds which reactivated CDKN2A expression in at least three independent experiments. Vehicle treated cells served as negative control, DAC treatment at indicated concentrations for three days as positive control, and water during RT reaction and during PCR as RT-PCR control. (PDF) [file pone.0044690.s002.pdf]
